# Striving for the perfect diet? The mediating role of perfectionism in the relationship between obsessive compulsive symptoms and traits of Orthorexia Nervosa

**DOI:** 10.1186/s40337-024-01032-w

**Published:** 2024-07-01

**Authors:** Maddy Greville-Harris, Laura Vuillier, Summer Goodall, Catherine V. Talbot, Cliona Walker, Katherine M. Appleton

**Affiliations:** Department of Psychology, Faculty of Science and Technology Poole House, Talbot Campus, Bournemouth University, Fern Barrow, Poole, Dorset, BH12 5BB UK

**Keywords:** Orthorexia nervosa, Disordered eating, Obsessive-compulsive symptoms, Perfectionism, Achievement striving, Evaluative concern, eating patterns

## Abstract

**Background:**

Orthorexia Nervosa (ON) is a disordered eating style involving an unhealthy obsession with ‘healthy’ or ‘clean’ eating. Its aetiology is still poorly understood and is not yet recognised in diagnostic manuals. While ON has been associated with Obsessive Compulsive (OC) symptoms and perfectionism, no study to date has looked at the relationship between OC symptoms and ON tendencies *via* perfectionism, or the influence of two facets of perfectionism in this relationship, namely evaluative concern and achievement striving. Examining the potential role of perfectionism helps to understand the aetiology of ON as well as inform potential treatments tailored specifically for ON and comorbid OC symptoms.

**Methods:**

In this cross sectional study, we tested 507 participants (*n* = 70 males, *n* = 69 at risk of ON) on questionnaires measuring OC symptoms, perfectionism and ON symptoms. We ran two mediation analyses to look at the overall relationship between perfectionism and OC and ON symptoms (Model 1) as well as the specific contribution of evaluative concern and achievement striving in the relationship between OC and ON symptoms (Model 2).

**Results:**

We found that perfectionism partially mediated the relationship between OC and ON symptoms. Specifically, we found that while achievement striving and evaluative concern were associated with OC symptoms, only achievement striving was significantly associated with ON symptoms, mediating the relationship between OC and ON symptoms.

**Conclusions:**

This study highlighted the role of one key facet of perfectionism (achievement striving) in the aetiology of ON. The role of achievement striving was indicated as a transdiagnostic construct explaining the link between ON and OC symptoms. These findings are discussed in terms of their implications for treatment models, specifically in terms of the potential role of targeting perfectionism in ON treatment.

**Supplementary Information:**

The online version contains supplementary material available at 10.1186/s40337-024-01032-w.

## Abstract (plain language summary)

Orthorexia Nervosa (ON) is a disordered eating style involving an obsession with extreme ‘healthy’ eating, causing the person distress and impairment. People with ON are more likely to have symptoms of Obsessive Compulsive Disorder (OCD), such as obsessive thoughts that are reduced by repetitive behaviours. There is also evidence that perfectionism (e.g. having very high personal standards) plays an important role in both ON and OCD.

Our study of 507 participants looked at whether two aspects of perfectionism: (1) concern about being evaluated negatively by others; ‘evaluative concern’, and: 2) striving for very high standards; ‘achievement striving’ were linked to higher levels of orthorexic and obsessive-compulsive symptoms. Our research also looked at whether these aspects of perfectionism explained the link between orthorexic and obsessive-compulsive symptoms.

We found that both aspects of perfectionism were linked to obsessive-compulsive symptoms. However, achievement striving specifically helped to explain the link between orthorexic and obsessive-compulsive symptoms. These findings suggest that treatment focusing on achievement striving might be particularly useful for people with ON, and for those who have both orthorexic and obsessive-compulsive symptoms.

**Supplementary Information:**

The online version contains supplementary material available at 10.1186/s40337-024-01032-w.

## Background

Orthorexia Nervosa (ON) is a disordered eating style involving an unhealthy obsession with ‘healthy’ or ‘clean’ eating, extreme control over diet, and subsequent impairment to areas such as social and emotional wellbeing [1]. Severe dietary rules in ON result in excessive avoidance of ‘unhealthy’ foods and distress when food rules are violated [2]. Despite the lack of recognition of ON in diagnostic manuals, ON is a recognised label used by those with symptoms [3] and is widely discussed on social media [4]. Obsessive Compulsive Disorder (OCD) is a mental health disorder characterised by persistent and intrusive thoughts, images, or urges (obsessions) that often trigger compulsions performed to alleviate distress or prevent a dreaded outcome [5]. There is increasing evidence of comorbidity between ON and OCD, with individuals with ON reporting high levels of OC symptoms [6]. Moreover, both ON and OCD feature a preoccupation with feared stimuli, as well as repetitive thoughts; in ON this may include intrusions related to food, contamination, and health, as well as compulsions around food preparation and consumption [6, 7]. Whilst a link between OC and ON symptoms is well established, the *mechanisms* underlying this relationship are little understood.

Perfectionism has been identified as a risk factor for OCD [8] and is linked to eating disorders (EDs), such as Anorexia Nervosa [9] and Bulimia Nervosa [10]. It is also thought to be a transdiagnostic factor that can explain the comorbidity between these disorders [11]. ON is increasingly argued to be an emerging ED [6], and perfectionistic beliefs (e.g., the belief that actions must be carried out perfectly) have been found to explain the need for ‘perfectly healthy’ dieting behaviours in ON [12]. However, no research has explored the mediating role of perfectionism in the relationship between OC and ON symptoms, which is the aim of the current study. Given the frequent overlap between these two conditions, understanding whether the crossover between ON and OC symptoms can be explained by common underlying constructs such as perfectionism can help inform our understanding of aetiology and treatment for ON and comorbid OC symptoms.

### The relationship between obsessive compulsive (OC) symptoms and disordered eating

There is considerable overlap between key features of OCD and EDs, with biological, genetic, and psychological factors common to both [13]. Given that EDs and OCD both feature a preoccupation with feared stimuli, as well as repetitive thoughts, there is some debate as to whether these conditions should lie on the same spectrum of disorders [11]. EDs and OCD also share a high lifetime comorbidity rate (35–44%; [14, 15]), with high levels of OC symptoms such as obsessive intrusions present for individuals with EDs [11, 13]. Whilst there is a substantial body of research exploring the relationship between OC symptoms and established EDs such as Anorexia Nervosa and Bulimia Nervosa [14], individuals with less well-defined EDs (such as Other Specific Feeding or Eating Disorder) tend to be excluded from such studies [11]. As such, whilst some research has highlighted shared characteristics in OCD and ON, such as intrusions related to food, contamination, and health, as well as compulsions around food preparation and consumption [6, 7], no studies have looked at what may be driving this relationship.

### The role of perfectionism

Perfectionism is conceptualised as comprising two key facets: (1) engaging in highly critical self-evaluation, also called evaluative concern, and: (2) setting unrealistic, high expectations, also called achievement striving [16]. Evaluative concern has historically been referred to as ‘maladaptive’ perfectionism and has been implicated as a transdiagnostic risk factor for psychopathology [17]. In contrast, achievement striving has historically been associated with positive mental health outcomes [18] although it has also been implicated in psychopathology, particularly EDs [19].

Perfectionism is highly prevalent in individuals with OCD [20] and has been identified as a risk factor [8] or predisposing trait [21] in the aetiology of OCD, as well as a contributing factor in maintaining OC symptoms [22]. Perfectionism is also a target for treatment interventions, with higher perfectionism being associated with poorer treatment outcomes for OCD [23], and changes in perfectionism highlighted as a potential key mechanism of change for reducing OC symptoms in therapy [20]. In terms of the key facets of perfectionism, evaluative concern has consistently been found to be elevated in individuals with OCD (e.g [24, 25]), and a recent meta-analysis [19] suggested it could be a key target for interventions for OCD. Although evaluative concern has been found to have a stronger relationship with OCD, research has also indicated elevated achievement striving in individuals with OCD compared with controls (see [19]).

Perfectionism has also been identified as a risk and maintenance factor in the development of EDs [19] and plays a key role in many models of EDs such as the three-factor model for BN [26] and the transdiagnostic model of EDs [27]. These models highlight the importance of striving for unattainable standards in EDs- specifically weight ideals- as a fundamental component in the development and maintenance of ED symptoms [26, 27]. Similarly to OCD, perfectionism has also been helpful in treatment for EDs. For example, interventions which target perfectionism have been found to reduce disordered eating [28], with CBT for perfectionism suggested to be as effective for reducing ED behaviours as ED-specific CBT [10]. Unlike other disorders such as OCD, where achievement striving is less strongly implicated [19, 24], both facets of perfectionism appear to play a fundamental role in the psychopathology of EDs, with elevated levels of both evaluative concern and achievement striving associated with ED symptoms [29].

A small number of studies also suggest a link between ON and perfectionism [30, 31]. For example, ON involves striving to adhere ‘perfectly’ to a healthy diet, following exacting dietary rules [30] and rigorous exercise regimes [32]. Perfectionistic self-presentation (specifically the need to portray a perfect self-image) is also associated with ON symptoms in individuals who engage in high levels of exercise [32, 33] and predicts ON symptoms over time [34]. No research, to the best of our knowledge, has looked at the role of the achievement striving and evaluative concern subtypes of perfectionism in ON, or the mediating role of perfectionism in explaining the overlap between On and OC symptoms.

### The current study

ON and OC traits are known to be linked [6, 35, 36], however the underlying mechanisms for this relationship are not yet clear. Existing evidence suggests that perfectionism is a key mediator in the relationship between OC and established EDs such as Bulimia Nervosa [37], binge eating behaviours [15] and eating pathology more generally [38, 39]. However, this relationship has not been explored in the context of ON. Inspired by existing findings for other EDs [29], the current study aims to understand whether perfectionism is associated with ON symptoms (including which of its two facets) as well as whether [31–34] perfectionism mediates the relationship between OC and ON symptoms [38].

This study will investigate three hypotheses using mediation analyses:

#### Hypothesis 1

(H1): In line with OCD literature [8, 20] higher levels of perfectionism traits will be associated with higher levels of OC symptoms (H1a). Specifically, we predict that both facets (evaluative concern and achievement striving) will be associated with higher levels of OC symptoms (H1b).

#### Hypothesis 2

(H2): In line with ED literature [19, 29] higher levels of perfectionism traits will be associated with higher levels of ON symptomatology (H2a). Specifically, we predict that both facets (evaluative concern and achievement striving) will be associated with higher levels of ON symptomatology (H2b).

#### Hypothesis 3

(H3): Similar to established models in OCD and ED research [38, 39], the relationship between OC symptoms and ON tendencies will be mediated by perfectionism (H3a). Specifically, given the importance of both facets of perfectionism in EDs [29], it is hypothesised that both evaluative concern and achievement striving will play a mediating role in the relationship between OC and ON symptoms (H3b).

## Methods

### Participants and recruitment

Participants were recruited via a University Psychology Student Research Participation database and through advertisement on social media. The data collection period took place between January 2022 and March 2023. Recruitment targeted students given the high prevalence of eating disorder symptomatology [40] and mental health conditions [41] in student populations. This approach was used to optimise recruitment of individuals scoring high on ON and OC symptomatology. Participating psychology students (83.5% of the sample) received course credits for participation. The recent existing mediation studies in this research area did not report effect sizes [15, 38] meaning that it was difficult to estimate a meaningful sample size for our study. However, according to Fritz & MacKinnon’s [42] guidelines for mediation analyses using percentile bootstrapping, the estimated sample size needed for 0.8 power for very small effects sizes (for path a and path b) was *n* = 558, with small-medium effect sizes (for path a and b) needing only a sample of *n* = 162. Our original sample size (*n* = 542) was close to the more conservative estimate. After removing 29 incomplete responses and 6 duplicate entries, 507 participants were included in the study. Sample demographics are outlined in Table 1.

**Table 1:** Key sample demographics

Demographic	Total sample (*n* = 507)
**Gender**, n (%)	
Male	70 (13.8)
Female	424 (83.6)
Non-binary	8 (1.6)
Other/prefer not to say	5 (1.0)
**Ethnicity**, n (%)	
White, White British or European	440 (86.8)
Black, African, Caribbean, or Black British	8 (1.6)
Asian or Asian British	20 (3.9)
Mixed, Multiple or Other Ethnic Group	39 (7.7)
**Age**, Mean (SD)	22.23 (8.7)

### Materials and procedure

This study used a cross sectional survey design. The study was approved by the lead author’s University Ethics committee (Ethics number 39,332). Participants were invited to take part in a survey hosted on Qualtrics XM. After indicating online consent, participants were directed to demographic questions (such as age, body mass index, and ethnicity) before completing a battery of validated questionnaires taking 20–30 min to complete.

Given the lack of consensus on the exact diagnostic criteria for ON [2] there is no single validated measure for ON symptomatology. Several measures such as the Dusseldorf Orthorexie Scale- English version (E-DOS [43]), and the Eating Habits Questionnaire (EHQ [44]), have been found to have good psychometric properties [45], but measure slightly different constructs. While the E-DOS is a unidimensional screening measure for orthorexic tendencies, the EHQ focuses on three aspects of knowledge, problems and positive feelings about extreme healthy eating. We decided to use both the aforementioned measures of ON; due to the fact that the E-DOS is a more widely used measure in published research and includes useful cut-off scores for participants ‘at risk’ of ON, we utilise the E-DOS for our main analysis, but also repeated our analysis using the EHQ as a confirmatory measure (see Supplementary Materials 1).

The questionnaire thus comprised measures of several potential risk factors for ON and included the Frost Multidimensional Perfectionism Scale- Brief (FMPS-B [17]), the Obsessive-Compulsive Inventory- Revised (OCI-R [46]), and the two measures of Orthorexia symptoms - The Dusseldorf Orthorexie Scale- English version (E-DOS [43]), and the Eating Habits Questionnaire (EHQ, [44]).

#### The Frost Multidimensional Perfectionism Scale- brief [FMPS-B]

The FMPS-B [17] consists of eight items measuring perfectionism, with four items used to measure each of the two subscales (evaluative concern and achievement striving). Items are scored on a 5-point scale from ‘1-strongly disagree’ to ‘5- strongly agree’ with the total score ranging from 8 to 40. Higher scores indicate higher levels of perfectionism. The FMPS-B has been found to have good psychometric properties [17], demonstrating very good internal consistency in our sample for the total scale (α = 0.86) and two subscales (evaluative concern: α = 0.82, achievement striving: α = 0.87).

#### The Obsessive-Compulsive Inventory- Revised [OCI-R]

The OCI-R [46] comprises 18 items measuring symptoms of OCD. Items are scored on a 5-point Likert scale from ‘0- not at all’ to ‘4-extremely’ with higher scores indicating greater levels of symptomatology. Summed scale scores of ≥ 21 suggest likely presence of OCD; in our sample, 234 participants (46.2%) scored within this range. The scale has been shown to have good internal consistency and excellent retest reliability [46], demonstrating excellent internal consistency in our sample α = 0.92.

#### The Dusseldorf Orthorexie Scale- English version [E-DOS]

The E-DOS [43] comprises ten items measuring orthorexic eating behaviours. Items are scored on a 4-point Likert scale from ‘1-this does not apply to me’ to ‘4- this applies to me’. Higher scores indicate greater ON symptomatology, with scores ≥ 25 indicating risk of ON and ≥ 30 indicating likely presence of ON. The E-DOS has been found to have good psychometric properties, with very good internal consistency and retest reliability [43]. The E-DOS also demonstrated very good internal consistency in our sample α = 0.87.

#### The Eating Habits Questionnaire [EHQ]

The EHQ [44] was used as a supplementary measure of ON symptoms (See Supplementary Material 1). This measure comprises 21 items with three subscales (Knowledge of healthy eating, Feeling positively about healthy eating, and Problems associated with healthy eating). Items are scored on a 4-point Likert scale from ‘1-false’ to ‘4-very true’, with the total score ranging from 21 to 84. The total score (rather than subscale scores) was used in this study. Higher total scores indicate higher levels of ON symptomatology. The EHQ has good psychometric properties [45] and demonstrated excellent internal consistency in our sample α = 0.92.

### Data analysis

All three hypotheses were investigated using two mediation analyses. The first analysis tested hypotheses H1a, H2a, and H3a, all focusing on an overall measure of perfectionism as the mediator, and the second analysis tested hypotheses H1b, H2b, and H3b, focusing on the facets of evaluative concern and achievement striving as mediators. Mediation analyses were carried out using the PROCESS macro for SPSS (Model 4) with bootstrapping sampling set at 5000 [47]. Our first analysis (model 1) looked at the overall relationship between perfectionism and OC and ON symptoms. As such, mean OC score (OCI-R) was entered as the predictor (X), mean perfectionism score (FMPS-B) as the mediator (M) and sum ON scores (E-DOS) as the Outcome variable (Y). Our second analysis (model 2) looked at the specific contribution of evaluative concern and achievement striving in the relationship between perfectionism and OC and ON symptoms. Therefore, in model 2, means for evaluative concern and achievement striving (FMPS-B subscale) were entered as mediators (M1 and M2 respectively) whilst the predictor (OC score) and outcome (ON tendencies) remained the same. Both models were rerun using the EHQ to replace the E-DOS as the outcome measure (Y) (See Supplementary Material 1).

## Results

### Descriptive data for main study measures

In our sample, *n* = 42 (8.3%) reported identifying with the ON label; *n* = 24 (4.7%) had been told they might have ON (including *n* = 10 who reported identifying with the label).The mean scores for our study measures and frequency of ED symptomatology are outlined in Table 2. 30 participants in our sample (5.9%) reported a past ED diagnosis, with nine participants (1.8%) reporting a current ED diagnosis. Based on E-DOS questionnaire cut-offs, *n* = 69 of the sample (13.6%) were in the ‘at risk for ON’ range (scoring ≥ 25 on the E-DOS), with *n* = 26 (5.1%) indicating ‘likely presence of ON’ on this scale (scoring ≥ 30 on the E-DOS) [43]. Of the 42 participants who self-identified with the ON label 28 (66.7%) also scored ‘at risk’ or ‘likely presence’ of ON on the E-DOS. However, of the 24 participants who reporting being told that they might have ON 9 (37.5%) scored in the ‘at risk’ or ‘likely presence’ on ON range on the E-DOS.

**Table 2:** Sample frequency and mean scores for main measures

Demographic	Total sample (*n* = 507)
**Questionnaire Scores**, Mean (SD)*	
Orthorexia symptoms (E-DOS)	17.59 (6.0)
Perfectionism total (FMPS-B)	23.50 (6.9)
Evaluative Concern (FMPS-B)	11.27 (4.0)
Achievement Striving (FMPS-B)	12.23 (4.0)
Obsessive-Compulsive symptoms (OCI-R)	22.27 (14.0)
**Eating Disorder (ED) Demographics**, n (%)	
Past ED	30 (5.9)
Current ED diagnosis	9 (1.8)
‘At risk’ for Orthorexia Nervosa (ON)**	69 (13.6)
‘Likely presence’ of ON***	26 (5.1)
Ever had ED who also report ‘risk’ for ON	14 (2.8)

*Measures are E-DOS- Dusseldorf Orthorexie Scale- English version [43]; *FMPS-B -Frost Multidimensional Perfectionism Scale- Brief* [17]; *OCI-R- Obsessive-Compulsive Inventory- Revised* [46]. ** ‘At risk’ for ON refers to scores ≥ 25 on the E-DOS. ***‘Likely presence’ of ON refers to scores ≥ 30 on E-DOS

### Model 1

**Fig. 1:**
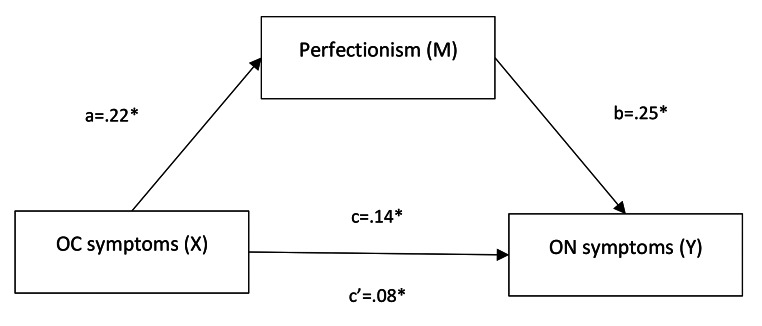
Mediation model (H1a, H2a, H3a): perfectionism as the mediator between OC symptoms and ON symptoms Note. regression coefficients for path a, b, c and c’ are presented in Fig. 1. *Denotes significance level of *p* < .001

As illustrated in Fig. 1, there was a significant relationship between OC symptoms and perfectionism (Path a: *b* = 0.22, *p* < .001; R^2^ = 0.20, F [1, 504] = 127.41, *p* < .001) and between perfectionism and ON tendencies (Path b: *b* = 0.25, *p* < .001), confirming H1a and H2a.

The total effect of OC symptoms on ON tendencies was significant when perfectionism was not included as a mediator (Path c: *b* = .14, *p* < .001; *R*^*2*^ = .11, *F* [1,504] = 56.35, *p* < .001). The direct effect of OC symptoms on ON tendencies was still significant, but reduced, when perfectionism was controlled for (Path c’: *b* = 0.08, *p* < .001). There was a significant indirect effect of perfectionism on the relationship between OC symptoms and ON tendencies (*b* = 0.06, CI [0.034, 0.080]) with a medium sized effect (completely standardized indirect effect = 0.129). This finding confirms H3a, with perfectionism acting as a mediator in the relationship between ON tendencies and OC symptoms.

### Model 2

**Fig. 2:**
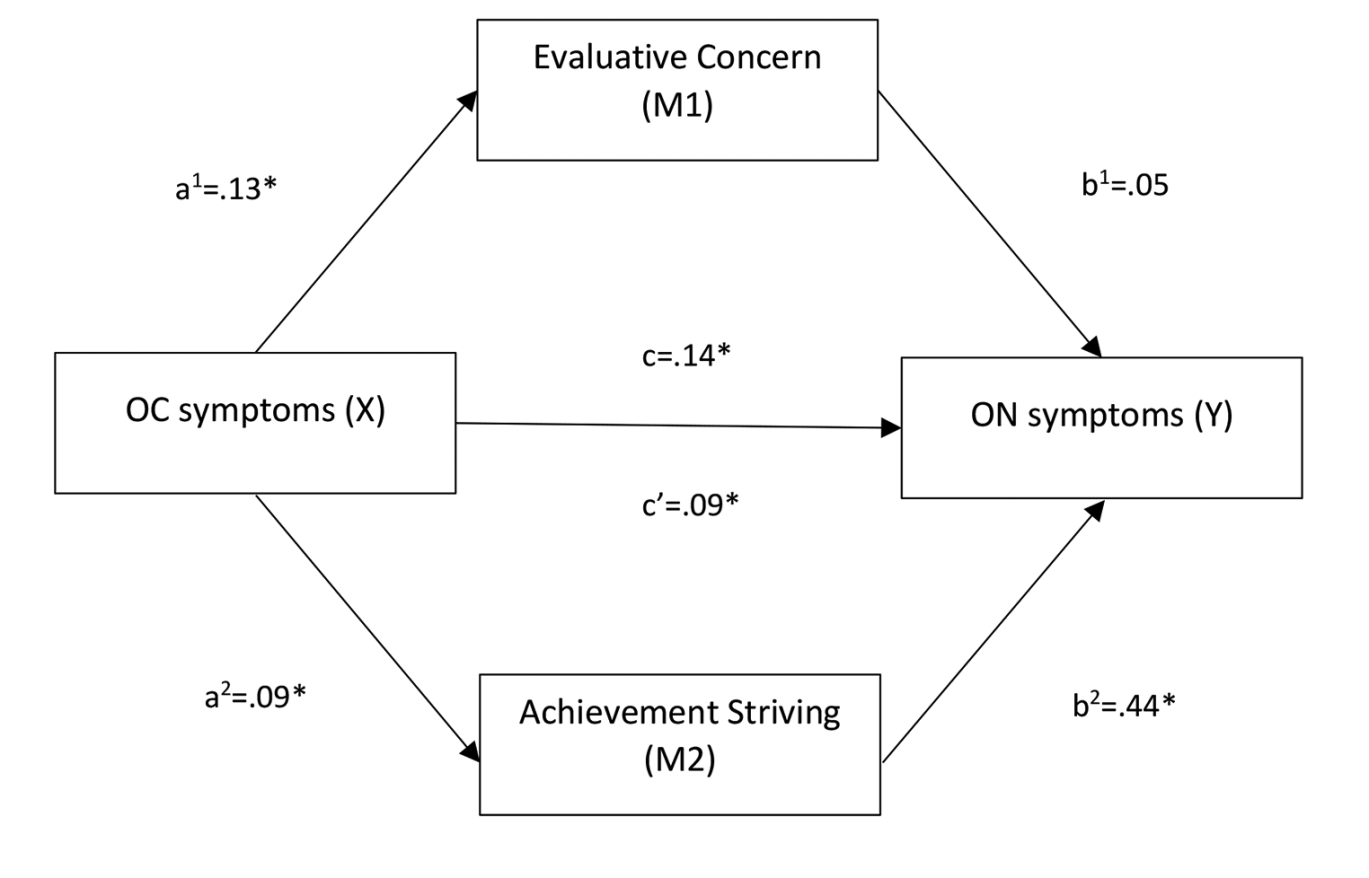
Mediation model (H1b, H2b, H3c): achievement striving (but not evaluative concern) mediates the relationship between OC symptoms and ON symptoms Note. regression coefficients for path a, b, c and c’ are presented here. *Denotes significance level of *p* < .001

As per Fig. 2, there was a significant relationship between OC symptoms and evaluative concern (Path a^1^: b = 0.13, *p* < .001; R^2^ = 0.20, F [1, 504] = 126.25, *p* < .001), as well as between OC symptoms and achievement striving (Path a^2^: b = 0.09, *p* < .001; R^2^ = 0.11, F [1, 504] = 59.61, *p* < .001) confirming H1b. There was no significant relationship between evaluative concern and ON tendencies (Path b^1^: *b* = 0.05, *p* = .508). There was, however, a significant relationship between achievement striving and ON tendencies (Path b^2^: *b* = 0.44, *p* < .001), partially confirming H2b.

The total effect of OC symptoms on ON tendencies was significant when evaluative concern and achievement striving were not included as mediators (Path c: *b* = .14, *p* < .001; *R*^*2*^ = .10, *F* [1,504] = 56.35, *p* < .001). The direct effect of OC symptoms on ON tendencies was still significant, but reduced, when the mediators were controlled for (Path c’: *b* = 0.09, *p* < .001).

There was a significant indirect effect of achievement striving on the relationship between OC symptoms and ON tendencies (*b* = 0.04, CI [0.025, 0.059]). This indicates a medium sized effect (completely standardized indirect effect = 0.095). In contrast, the indirect effect of evaluative concern was not significant in the model (*b* = 0.006, CI [-0.014, 0.027], completely standardised effect = 0.015). Overall, achievement striving was a mediator in the model, where evaluative concern was not, only partially confirming H3b.

Both mediation analyses were also run with the EHQ as the measure of ON symptoms (Y). These models yielded the same pattern of results (see Supplementary Material 1), also partially supporting our hypotheses and strengthening our findings.

## Discussion

In this paper, we aimed to elucidate the observed relationship between ON and OCD, by looking at the mediating role of perfectionism. We tested 507 participants on a battery of questionnaires measuring ON symptoms, OC traits and perfectionism. We hypothesised that higher levels of perfectionism would be associated with higher levels of OC symptoms (H1a) and greater levels of ON symptomatology (H2a). More specifically, we hypothesised that both facets of perfectionism (evaluative concern and achievement striving) would be associated with higher levels of OC symptoms (H1b) and greater ON symptomatology (H2b [19]). We also hypothesised that perfectionism (H3a) (and perfectionism facets of evaluative concern and achievement striving (H3b)) would mediate the relationship between OC and ON symptoms in line with similar models for other EDs (e.g [38, 39]). These hypotheses were partially supported; perfectionism (and specifically achievement striving) mediated the relationship between ON and OC symptomatology. The specific relationships in these mediation models are discussed in more detail below.

### The relationship between perfectionism and OC symptoms

In this study, higher levels of perfectionism (and higher evaluative concern and achievement striving individually) were associated with greater levels of OCD symptomatology (as hypothesised in H1a and H1b). This finding is in line with existing research which has previously highlighted the prevalence of perfectionism in individuals with OCD [20] and implicated perfectionism as a key belief domain implicated in OCD [48]. While a meta-analysis has indicated the role of evaluative concern in particular in individuals with OCD [19], our study is also in line with studies suggesting a relationship between OCD and achievement striving [19, 24, 49].

### Perfectionism and ON: the role of achievement striving, but not evaluative concern

As predicted (H2a), higher levels of perfectionism were associated with higher levels of ON symptoms in our study. When focusing on perfectionism facets (H2b), as expected, higher levels of ON symptoms were associated with higher levels of achievement striving. Thus, similar to other EDs [26, 27] striving for high standards may be important in the aetiology of ON, with achievement striving playing a key role in ON symptoms. While existing research suggests a link between ON and perfectionism more generally [30, 31] this finding suggests that perfectionistic high standards and goal setting in particular, may be linked to ON symptoms such as striving for the ‘perfectly’ healthy diet and exacting dietary rules.

A novel finding from this study was the lack of association between ON symptoms and evaluative concern. There are several explanations for why ON might differ from other established EDs in this respect. First, ON is by definition more focused on health than weight concerns. Concerns about evaluation from others may be less relevant when dietary motivation is related to health, given that motivation may be internally focused (e.g. to feel healthy) rather than externally focused (e.g. to look a certain way to others). While it is recognised that the motivations for ON are complex, and that ideas about health and weight are often conflated in the context of ON [50], there is some evidence that some ON behaviours are indeed internally motivated [51]. For example, Oberle and colleagues [51] explored exercise motivations in the context of ON; individuals with higher ON symptoms reported that their exercise motivation was due to internal factors such as the value and enjoyment of exercise, and the guilt of missing a fitness session. External motivations (such as being told to exercise by others) was not related to ON symptomatology. Moreover, individuals high in ON symptoms have conceptualised ‘healthy eating’ as related to healthy states of mind, working at peak performance, removing pathogens from their body and feeling in control as well as adhering to body ideals [50]. Arguably, all of these conceptualisations except body ideals are related to internal rather than external motivations, suggesting that concern about evaluation from others may perhaps be less relevant in the pursuit of the excessive healthy eating and exercise related to ON. Future research would be useful to explore the validity of this potential explanation.

Second, concern about evaluation from others and self-criticism may be less relevant in the context of ON, where symptoms have been associated with higher levels of narcissism and feelings of superiority to others [52]. Pursuing healthy eating in ON is considered ego-syntonic, with individuals high in ON symptoms believing their obsessions around food to be appropriate and desirable [53]. The pursuit of healthism -whereby health is considered an individual’s responsibility and moral obligation- is valued by Western societies, with pursuing health considered a sign of emotional strength and goodness [54]. Individuals who exhibit higher ON traits may therefore be less concerned by evaluation from others due to the social acceptability of their ‘healthy eating’ choices, or a sense of superiority, achievement and appropriateness of their health choices comparative to others. Indeed, research exploring narratives of ON recovery on Instagram has highlighted how ON behaviours are normalised and idealised, serving as a barrier to ON recognition and recovery [3]. Moreover, validated orthorexia measures focus on positive comparisons or superiority, including items such as ‘I like that I pay more attention to healthy nutrition than other people’ (E-DOS [43]) and ‘My eating habits are superior to others’ (EHQ [44]). More research is needed to clarify the role of sense of superiority in the context of ON, and whether or not perceived superiority mitigates against experiences of evaluative concern in those with ON symptoms.

Third, it is likely that there is a complex and close relationship between both facets of perfectionism; indeed in our data evaluative concern and achievement striving scores were moderately correlated, suggesting that high scores on evaluative concern were related to corresponding high scores on achievement striving. It could be that both perfectionism facets play a key role in the context of ON symptomatology, albeit with achievement striving explaining more variance in our data. Future research exploring the interrelated role of these facets in predicting ON traits would be useful.

### Perfectionism as a mediator between OC and ON symptoms

In line with our hypothesis (H3a), and established models in OCD and ED research [38, 39], the relationship between OC symptoms and ON tendencies was partially mediated by perfectionism in our study. However, contrary to our hypothesis (H3b), due to the lack of association between evaluative concern and ON, only achievement striving (and not evaluative concern) mediated this relationship. This suggests that perfectionism (specifically the achievement striving facet) may serve as a transdiagnostic construct helping to explain the comorbidity between symptoms of ON and OCD. This finding is in line with previous research, where the transdiagnostic role of perfectionism has been found to partly explain the overlap between eating pathology and OC symptoms [15, 37–39].

### Study implications

There is increasing research indicating the promise for perfectionism-based interventions for OC symptoms [55] and eating pathology [28] as well as transdiagnostically across disorder profiles [22, 56]. Our research suggests that ON too might benefit from treatments which target this transdiagnostic construct. However, many current CBT-based interventions for perfectionism target the reduction of evaluative concern, but not achievement striving [19, 22], while our study suggests that specific focus on achievement striving would be needed within the context of ON. Interventions targeting both facets of perfectionism have been developed specifically for other EDs, with a recent meta-analysis highlighting that perfectionism interventions significantly reduced perfectionism and disordered eating with large treatment effect sizes [28]. For Anorexia Nervosa specifically, a six week group-based cognitive behavioural perfectionism intervention has been developed and piloted in individuals in an inpatient setting [57, 58]; participants reported that the intervention helped them to develop awareness of perfectionism, recognise its negative impact and challenge perfectionist thoughts and behaviours. This intervention targeted both achievement striving and evaluative concern, and as such, our study suggests that aspects from this established intervention to reduce achievement striving (through striving for excellence rather than perfection), may be particularly relevant in the context of ON treatment. However, it should be noted that the ED models outlining the role of perfectionism in disorders such as Anorexia Nervosa [27] and Bulimia Nervosa [26] may not directly translate to individuals with ON, so further exploration of the role(s) of achievement striving and evaluative concern is needed in the context of ON.

### Strengths, limitations and future research

This study is the first to date to explore the potential mediating role of perfectionism in the relationship between OC and ON symptoms. Nevertheless, there are several limitations to consider. First, given the lack of diagnostic criteria for ON, identifying individuals scoring ‘high’ in ON symptomatology is complex and not yet clearly defined. Whilst some scales to measure ON symptoms such as the ORTO-15 show poor reliability [45], we used two scales with strong psychometric properties (E-DOS [43]; EHQ [44]) and found the same associations between perfectionism, ON and OC for both. We note however, that the association between ON and achievement striving was larger when measured with the EHQ (b = 0.98) compared with the E-DOS (b = 0.44). This may be due to the EHQ focusing more on dietary satisfaction than the E-DOS, including items such as ‘I feel great when I eat healthily’. Thus, until we have a clear diagnostic tool for ON, it is important to confirm findings with more than one measure of ON.

Second, our cross-sectional design allowed us to look for correlational associations, but not to infer the temporal causal links between perfectionism and ON/OC symptoms. Mediational analyses were designed to deal with longitudinal data and thus future studies are needed to confirm these findings using at least two timepoints. While existing literature suggests that perfectionistic traits serve as a risk factor for the development of EDs, and that OC symptoms often predate ED symptomology, there is a lack of longitudinal studies to support these premises [59]. Thus, future longitudinal prospective studies would be useful, in order to establish the temporal relationships between perfectionist traits, ON and OC symptomology.

Third, our sample was predominantly White/European (86.8%) and female (83.6%); minority ethnic groups, male, and non-binary individuals could thus be better represented in future work. Despite this limitation, a relative strength in our recruitment was in targeting a large, predominantly student population (83.5%) given the high prevalence of mental health conditions [41] and eating disorder symptomatology [40] in such populations. Indeed, ON symptomatology was relatively high in our sample, with a significant proportion (13.6%) scoring ‘at risk’ for ON, 8.3% identifying with the ON label and 5.1% scoring above the cutoff for ‘likely presence’ of ON. This finding is in line with a recent largescale meta-analysis of disordered eating in university students [60] which estimated that nearly 20% of students reported disordered eating. It also aligns with recent ON research suggesting the ‘likely presence’ of ON in university students ranges from 2.3 to 8.4% [61].

Our sample also displayed a high incidence of OC symptoms, with 46.2% scoring within the ‘likely presence of OCD’ range on the OCI-R. While high incidence of OC symptoms, have been reported in young people in other recent studies [62, 63], this incidence could reflect: (1) the impact of the pandemic in terms of increased incidence of mental health issues for young people [64]: (2) the sensitivity of the scale OCI-R measure in picking up on handwashing behaviours/ contamination fears, which have perhaps become more normalised as a result of the pandemic. Our data were collected after the 2019 Covid-19 pandemic at a time when the incidence of mental health difficulties for students in the UK was known to be high; indeed the Cibyl Student National Health Survey [65] estimated that over 80% of students nationally were experiencing mental health difficulties at this time. While we used continuous scale ratings, rather than cut-offs for our analyses, future research might consider the sensitivity of measures of OC symptomatology in light of the increased incidence in mental health/OC symptoms.

## Conclusions

This study was the first to explore whether the relationship between ON and OC symptoms can be explained via the transdiagnostic role of perfectionism. Specifically, the role of two facets of perfectionism (achievement striving and evaluative concern) were investigated. We found that perfectionism partially mediated the relationship between OC and ON symptoms as hypothesised. However, while achievement striving and evaluative concern were associated with OC symptoms, only achievement striving was associated with ON symptoms. Thus, only achievement striving was implicated as a transdiagnostic construct explaining the relationship between ON and OC symptoms. This study highlighted the potential role of achievement striving in the aetiology of ON, suggesting that this facet of perfectionism may be a useful target for ON interventions and for comorbid ON-OC symptoms.

### Electronic supplementary material

Below is the link to the electronic supplementary material.

Supplementary Material 1

## Data Availability

The datasets generated and analysed during the current study are available in Bournemouth University’s Online Research Data Repository BORDaR: 10.18746/bmth.data.00000335.

## Abbreviations

E-DOS: Dusseldorf Orthorexie Scale-English version
ED: Eating Disorder
EHQ: Eating Habits Questionnaire
FMPS-B: Frost Multidimensional Perfectionism Scale-Brief
OC: Obsessive Compulsive
OCD: Obsessive Compulsive Disorder
OCI-R: Obsessive Compulsive Inventory-Revised
