# Supplementary material for: Striving for the perfect diet? The mediating role of perfectionism in the relationship between obsessive compulsive symptoms and traits of Orthorexia Nervosa
Source: J Eat Disord. 2024 Jul 1;12:91. doi: 10.1186/s40337-024-01032-w (PMC11218215; doi:10.1186/s40337-024-01032-w)
Supplement: Supplementary file 1 — Supplementary Material 1 [file 40337_2024_1032_MOESM1_ESM.docx]

**Supplementary Material 1- Mediation Models using the Eating Habits Questionnaire (EHQ) as a supplementary measure of ON.**

**Model 1:**

As illustrated in Figure 1, there was a significant relationship between OC symptoms and Perfectionism (Path a: b = .22, p < .001; R^2^ = .20, F [1, 503] = 127.23, p < .001) and between Perfectionism and ON tendencies (Path b: *b* = .43, *p* < .001), confirming H1a and H2a.

The total effect of OC symptoms on ON tendencies was significant when Perfectionism was not included as a mediator (Path c: *b* = .26, *p* < .001; *R^2^* = .12, *F* [1,503] = 68.03, *p* < .001). The direct effect of OC symptoms on ON tendencies was still significant, but reduced, when Perfectionism was controlled for (Path c’: *b* = .17, *p* <.001). There was a significant indirect effect of Perfectionism on the relationship between OC symptoms and ON tendencies (*b* = .10, CI [.060, .137]) with a medium sized effect (completely standardized indirect effect = .126). This confirms H3a, with perfectionism acting as a mediator in the relationship between ON tendencies and OC symptoms.

O-C symptoms (X)

ON symptoms (Y)

Perfectionism (M)

a=.22*

b=.43*

c’=.17*

c=

c

c=.26*

c=

c

*Note. b values for path a, b, c and c’ are presented in Figure 1.* *Denotes significance level of p<.001

**Figure 1.**

*Mediation model (H1a, 2a, 3a): Perfectionism as the mediator between OC symptoms and ON symptoms.*

**Model 2:**

As per Figure 2, there was a significant relationship between OC symptoms and Evaluative Concern (Path a^1^: b = .13, p < .001; R^2^ = .20, F [1, 503] = 125.96, p < .001), as well as between OC symptoms and Achievement Striving (Path a^2^: b = .09, p < .001; R^2^ = .11, F [1, 503] = 59.65, p < .001) confirming H1b. There was no significant relationship between Evaluative Concern and ON tendencies (Path b^1^: *b* = -.16, *p* =.195). There was, however, a significant relationship between Achievement Striving and ON tendencies (Path b^2^: *b* = .98, *p* < .001), partially confirming H2b.

The total effect of OC symptoms on ON tendencies was significant when Evaluative Concern and Achievement Striving were not included as mediators (Path c: *b* = .26, *p* < .001; *R^2^* = .12, *F* [1,503] = 68.03, *p* < .001). The direct effect of OC symptoms on ON tendencies was still significant, but reduced, when the mediators were controlled for (Path c’: *b* = .19, *p* <.001).

There was a significant indirect effect of Achievement Striving on the relationship between OC symptoms and ON tendencies (*b* = .09, CI [.059, .130]). This indicates a medium sized effect (completely standardized indirect effect = .121). In contrast, the indirect effect of Evaluative Concern was not significant in the model (*b* = -.021, CI [-.058, .014], completely standardised effect =-.028). Overall, Achievement Striving was a mediator in the model, where Evaluative Concern was not, only partially confirming H3b.

O-C symptoms (X)

ON symptoms (Y)

Evaluative Concern (M1)

a^1^=.13*

b^1^=-.16

c’=.19*

Achievement Striving (M2)

a^2^=.09*

b^2^=.98*

c=.26*

*Note.* b values for path a, b, c and c’ are presented here. *Denotes significance level of p<.001.

**Figure 2.**

*Mediation model (H2): Achievement Striving (but not Evaluative Concern) mediates the relationship between OC symptoms and ON symptoms.*
